# Time rescaling reproduces EEG behavior during transition from propofol anesthesia-induced unconsciousness to consciousness

**DOI:** 10.1038/s41598-018-24405-z

**Published:** 2018-04-16

**Authors:** S. Boussen, A. Spiegler, C. Benar, M. Carrère, F. Bartolomei, P. Metellus, R. Voituriez, L. Velly, N. Bruder, A. Trébuchon

**Affiliations:** 1 Department of Anesthesiology and Intensive Care, CHU Timone, Assistance Publique Hôpitaux de Marseille, Aix Marseille Université, 264 rue Saint-Pierre, 13005 Marseille, France; 2 Aix Marseille Université, IFSTTAR, LBA UMR_T 24, 13916 Marseille, France; 3 Institut de Neurosciences des Systèmes - Inserm UMR1106 - Aix-Marseille Université - Faculté de Médecine, 27, Boulevard Jean Moulin, 13005, Marseille, France; 4 Clinical Electrophysiology Department, CHU Timone, Assistance Publique Hôpitaux de Marseille, Aix Marseille Université, 264 rue Saint-Pierre, 13005 Marseille, France; 5 Neurosurgery Department, CHU Timone, Assistance Publique Hôpitaux de Marseille, Aix Marseille Université, 264 rue Saint-Pierre, 13005 Marseille, France; 6 Laboratoire Jean Perrin-UMR 8237 CNRS Université Pierre et Marie Curie, 75005 Paris, France; 7 Institut des Neurciences de la Timone, CNRS UMR1106 - Aix-Marseille Université - Faculté de Médecine, 27, Boulevard Jean Moulin, 13005, Marseille, France

## Abstract

General anesthesia (GA) is a reversible manipulation of consciousness whose mechanism is mysterious at the level of neural networks leaving space for several competing hypotheses. We recorded electrocorticography (ECoG) signals in patients who underwent intracranial monitoring during awake surgery for the treatment of cerebral tumors in functional areas of the brain. Therefore, we recorded the transition from unconsciousness to consciousness directly on the brain surface. Using frequency resolved interferometry; we studied the intermediate ECoG frequencies (4–40 Hz). In the theoretical study, we used a computational Jansen and Rit neuron model to simulate recovery of consciousness (ROC). During ROC, we found that *f* increased by a factor equal to 1.62 ± 0.09, and *δf* varied by the same factor (1.61 ± 0.09) suggesting the existence of a scaling factor. We accelerated the time course of an unconscious EEG trace by an approximate factor 1.6 and we showed that the resulting EEG trace match the conscious state. Using the theoretical model, we successfully reproduced this behavior. We show that the recovery of consciousness corresponds to a transition in the frequency (f, δf) space, which is exactly reproduced by a simple time rescaling. These findings may perhaps be applied to other altered consciousness states.

## Introduction

General anesthesia (GA) is an example of a reversible manipulation of consciousness, which is performed every day in hospitals around the world. While the mechanisms that underlie the effects of anesthetics become clearer at the cellular level^1,2^, the effects of anesthetics on brain activity at the scale of functional neural networks are still debated, which leaves space for several competing hypotheses^3^. GA shares many common pathways with sleep, specifically with respect to the loss and recovery of consciousness, but it also displays specific electrophysiological features that are related to drug effects. The changes in the EEG during sleep or anesthesia are empirically well described but their function and generation are still unknown^4^. The loss of consciousness induced by the widely used anaesthetic propofol is associated with an increase of power in the low-frequencies of the EEG and the emergence of strong and highly structured rhythmic activities in the EEG^5–7^ corresponding to frontal alpha oscillations (8 to 13 Hz)^5^. Many anesthetics share the same effects^8^. Moreover, for some authors, the characteristic changes in the EEG during GA are similar to those observed in the first stages of sleep^9,10^. A consistent explanation for these findings is missing and the behavior is still debated, which has led to different models^3,11–14^.

In this article, we recorded Electrocorticography (ECOG) after stopping the infusion of anaesthetics for brain surgery requiring functional testing in awake patients. We investigated the reorganization of dynamics in local frontal networks on the brink of regaining consciousness and tracked the changes in cerebral activity during the recovery of consciousness (ROC). We analysed the data using time-frequency analysis and we found a scaling factor between the conscious and the unconscious state. This scaling factor was demonstrated by formally time compressing an unconscious state and compared the obtained artificial state with a true conscious state. These results were interpreted with a theoretical framework that allows for an identification of the mechanism underlying the change in the dynamics of the ECoG.

## Results

### Clinical and patient’s settings

We selected six patients from a database of 25 patients. The patients had a total of five glial tumors and one cavernoma. We recorded ECoG signals for the six patients who underwent intracranial monitoring during awake surgery for the treatment of cerebral tumors in functional areas of the brain^15,16^. The ECoG grid was placed on the brain cortical surface after opening the skull under GA using total intravenous anesthesia with propofol and remifentanil. We recorded the ECoG throughout the cessation of anesthesia up to the point of the surgical removal of the tumour. Therefore, we recorded the transition from unconsciousness (state U) to consciousness (state C) directly on the brain surface. ROC was defined either by the spontaneous opening of the patient’s eyes or the patient’s response to a simple order. ROC occurred over a broad time range, 6 minutes to 21 minutes, after stopping the anesthesia supply (with a median time of 10 minutes). For the sake of homogeneity across patients, we focused on the frontal electrodes because (i) the underlying cortical region was free of tumorous tissue for all six patients and (ii) the frontal lobe has been found to be essential for consciousness. The number of electrodes per patient ranged from 21 to 40 (ESM 1, supplementary files).

**Figure 1:**
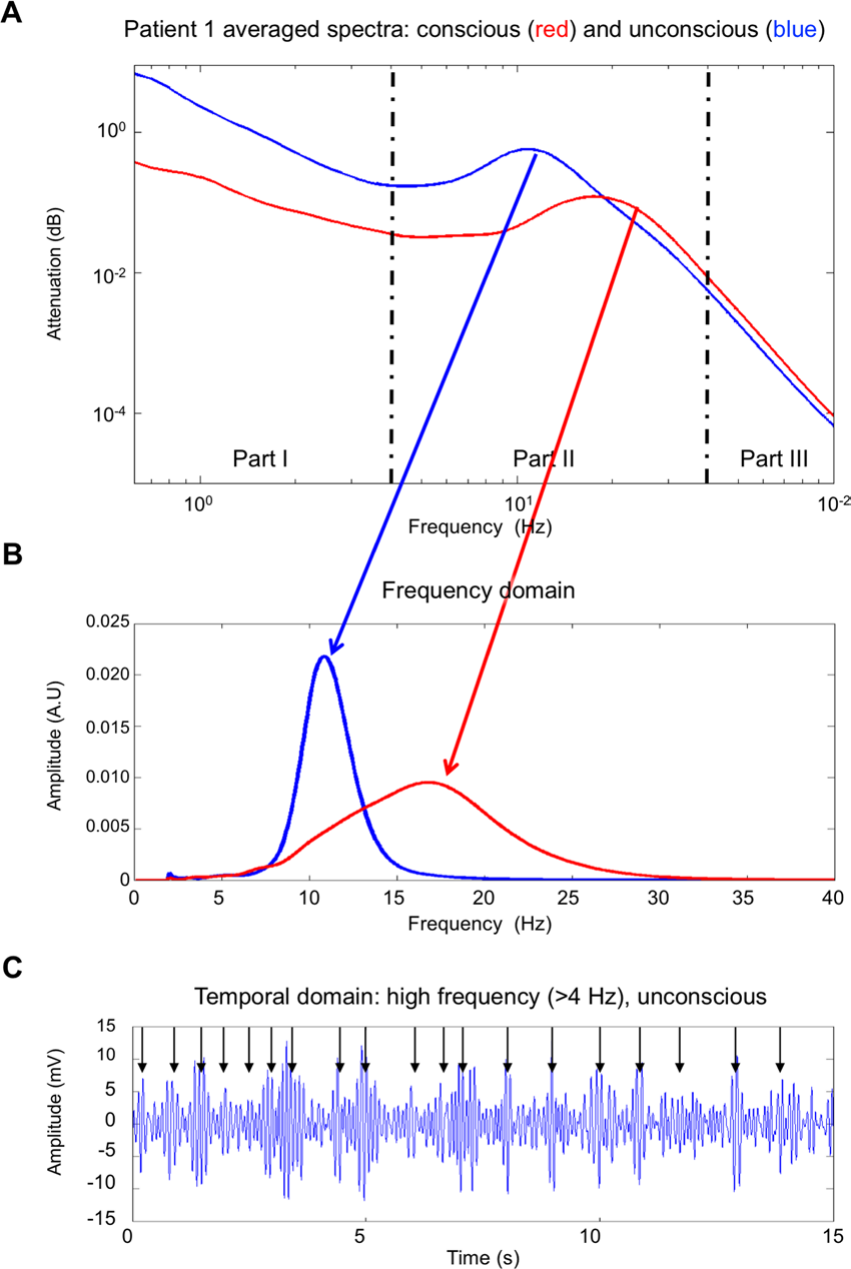
Typical ECOG frequency and temporal behavior during anesthesia and after recovery **(A)**. Spectral behavior: log power spectral density during unconsciousness (blue) and after ROC (red). These spectra were obtained from the same EEG channel recorded for 100 s in each condition. The spectrum shifts to 18 Hz and broadens when low frequencies (delta band) decrease. (**B)** power in the 4–40 hz region during unconsciousness (blue) and consciousness. 1/f^m^ background was removed using coarse grain analysis. (**C**) Temporal characteristics of pass band filtered (4–40 Hz) ECoG during unconscious state (blue). The time series display a succession of oscillatory pulses (arrows*)*.

### Time frequency analysis

ECoG provides enhanced spatial specificity compared with non-invasive electrophysiological measurement techniques, which allows for better discrimination between cortical sources. Typical ECoG recordings during GA (cerebral state U) and after ROC (cerebral state C) show that signal content differs at different scales (Fig. 1-A).

- The time series during GA and after ROC are dominated in the intermediate frequencies (Region II: 4–40 Hz) by a regular rhythm that accelerates from 11 Hz during GA to 18 Hz after ROC in the awake state (Fig. 1-B).
- The frequency spectrum of the typical ECoG signal clearly indicates power-law behaviors 1/*f*^*m*^ in the low-frequency range of (Region I: 0 < *f* < 4 Hz) and at high frequencies (Region III > 40 Hz) (Fig. 1-A). This behavior is consistent with the findings in the literature for ECoG measurements^17,18^.

The different part of the spectrum describes two very different patterns: an arrhythmic part that translate into a 1/*f*^*m*^ behavior (region I and III of Fig. 1) and bell-shaped spectrum that straddle between 5 and 40 Hz (Region II of Fig. 1). Study of the spectrum reveals that main changes occur in region II during ROC. These changes are an acceleration of the central emission frequency f with a widening of the spectrum δf.

**Figure 2:**
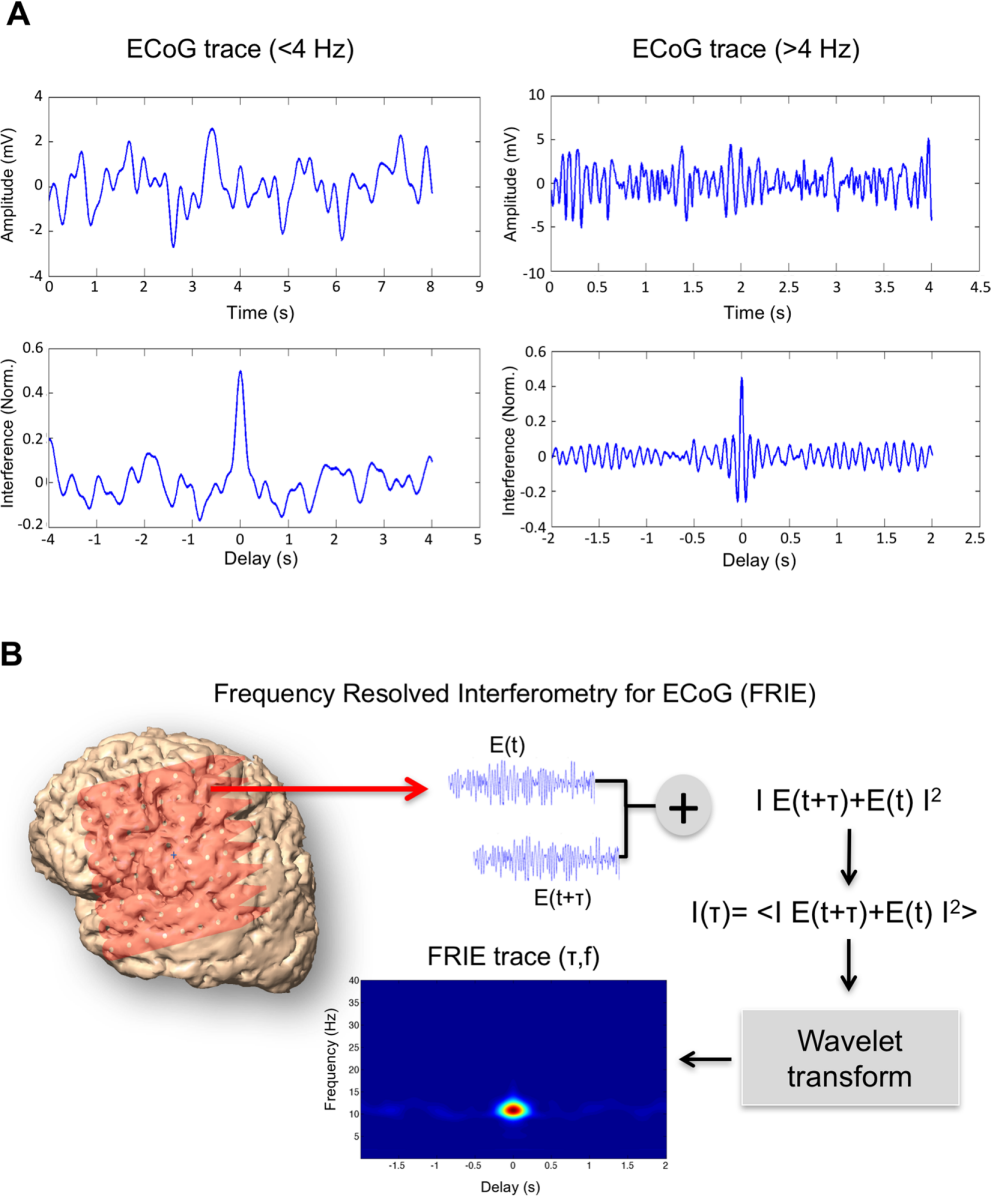
Principle of signal interferometry and example. (**A**) Interference of low frequencies (<4 Hz-left) and high frequencies (>4 Hz –Right). The upper graphs show the EEG trace and in the lower graphs the corresponding interferogram. The right interferogram shows interference fringes that are not present in lower frequency (left panel). (**B**) Frequency-Resolved Interferogram (FRIE) technique principle: the ECoG trace is delayed, added to itself and then averaged. After computing the interferogram, it is resolved in the frequency domain by wavelet transform obtaining a trace that resolves the interferogram in both time and frequency domain.

The patient’s state in region II is therefore described by two a priori independent variables f, δf and two dependent variables Q = f/ δf and τ the coherence length. Indeed, Q depends on f and δf and τ depends directly on δf. To compute the f and δf, we performed a time-frequency analysis of an interferogram of the ECoG. This technic allows separating 1/f^m^ and the oscillation behaviors. We therefore computed the frequency resolved interferogram of the ECoG signals (FRIE) that represents together the central frequency, the spectral and temporal width of the signal. The advantage of this technic is to show that nature of cerebral waves is different: Fig. 2 displays FRIE traces of an unconscious and conscious patient and their temporal structures. FRIE shows a strong coherent structure around 11 Hz in the unconscious state and 18 Hz in the conscious state. The FRIE technique allows us to compute a ‘coherent spectrum’ that is different from the total spectrum because the arrhythmic part does not contribute to the interference. Figure 2 shows the FRIE trace of the delta band and higher frequency band. Low frequencies do not have interference fringes, whereas high frequencies show regular fringes vanishing with time delay.

**Figure 3:**
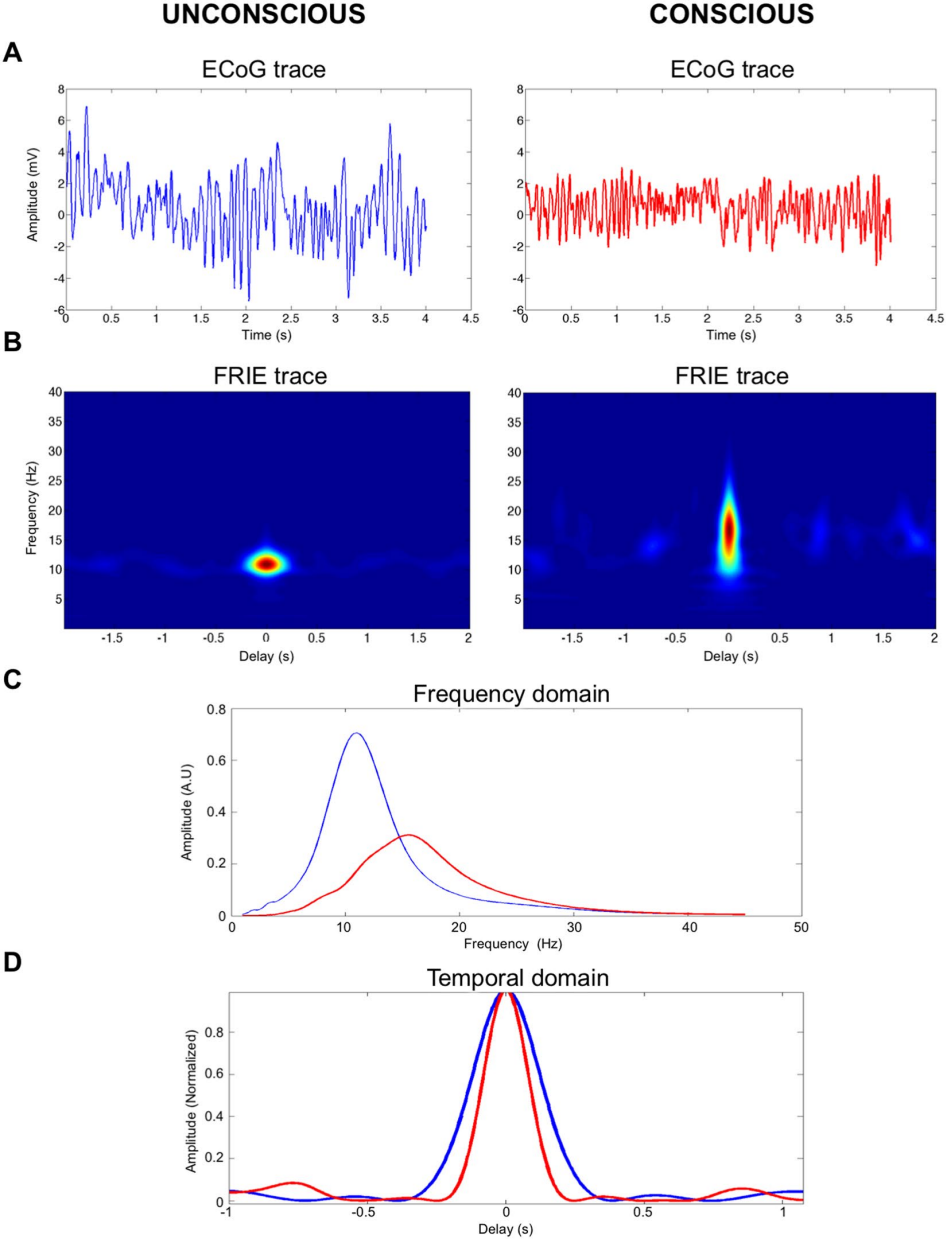
Exemple of ECOG trace during unconsciousness and after recovery of consciousness and the resulting FRIE. (**A**) EEG trace unconscious (blue) and conscious (red) (unfiltered). (**B**) Corresponding Frequency-Resolved Interferogram (FRIE) traces of the two states U and C: After ROC, the trace is compressed in the time domain and widened in frequency domain. (**C** and **D**) Corresponding frequency and time domain of the two states.

From FRIE trace we computed spectral and temporal characteristics of the intermediate frequencies. In principle, f and δf should evolve independently, but this is not the case. As shown in Fig. 3, the change of state appears in the FRIE trace as compression in the time domain and widening in the frequency domain. The transition during ROC squeezes the oscillation in a very similar manner to the notion of “squeezed coherent sate” of quantum -mechanics. That effect arises when there is an uncertainty relation between two observables^19^. Squeezed quantum states are due to the fact that a small uncertainty in one observable leads to a large uncertainty in another. Applied to the ROC process, the spectral widening leads to time shortening suggesting a scaling factor.

**Figure 4:**
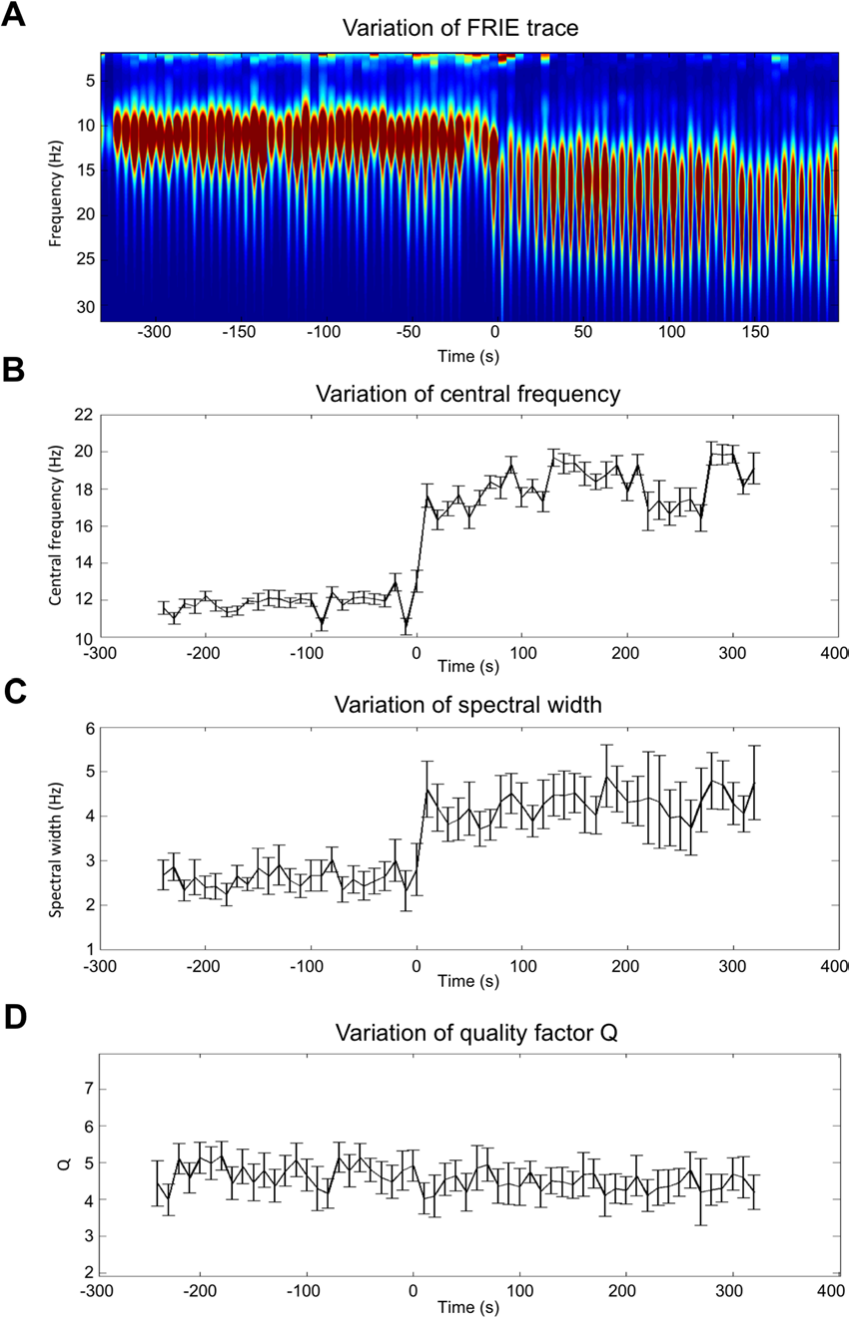
Evolution of frequency content and spectral width during the process of recovery of consciousness after general anesthesia in one patient. Upper graph (**A**) is the time-frequency representation obtained using wavelet analysis of repetitive FRIE (2 s FRIE trace placed end to end). Middle graph (**B**) is the change in time of the carrier frequency, and (**C**) is the width of the emission (Full width at half maximum). Lower graph (**D**) is the change of Q the quality factor defined by f/δf.

We tracked the central frequency and pulse FWHM changes with time during ROC process (Fig. 4-A shows the time–frequency change of a patient followed with a repetitive FRIE trace). Central frequency and spectral full width at half maximum (FWHM) evolution, as shown in Fig. 4B and C, is a sigmoid that has two states: The mean carrier frequency jumped, while the mean FWHM broadened. During the ROC process, the EEG changes more or less rapidly across patients: The transition time (the time needed for the oscillation to stabilize and its frequency to remain approximately constant) f was ranging from 77 s to 673 s across patients.

**Figure 5:**
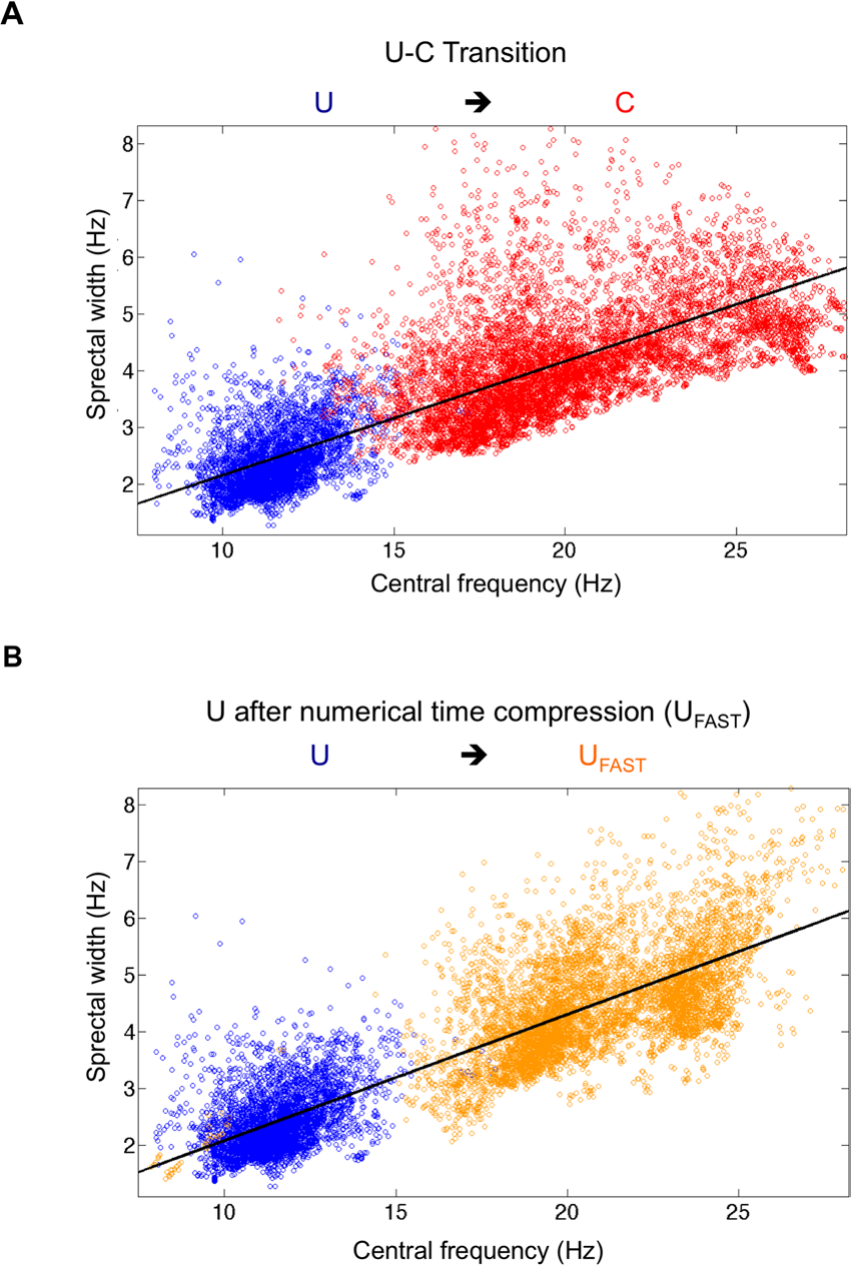
Representation of the consciousness state in the f-δf space for all 6 patients. (**A**) Blue dots represent frequency-spectral widths (FWHM) state during unconsciousness, the red dots are f_0_, δf couples after ROC. We obtained two distinct clouds. Frequency and spectral width are dependant and follow a linear relation symbolized by the black line (slope 0.20, correlation 0.84) (**B**) blue dots at the same U state than A figure, orange dots are the results of the time compression of the blue dots. The black line represents the linear regression (slope 0.22 correlation 0.88).

During ROC, the activity jumps from the unconscious state U: (*f*_0_ = 11.6 ± 0.6 Hz, *δf* = 2.7 ± 0.1 Hz) to the conscious state C: (*f*_0_ = 18.8 ± 0.4 Hz, *δf* = 4.4 ± 0.2 Hz) while *Q* remains constant (Q = 4.4 ± 0.1 vs Q = 4.4 ± 0.1 NS). Transposition of this effect in the audible range may be found as a supplementary file. We found that *f* increased by a factor equal to 1.62 ± 0.09, and *δf* varied by the same factor (1.61 ± 0.09). There was no statistically difference between the 2 ratios (P = 0.8). We showed that there was a linear correlation between *f* and *δf* and the relation was *δf* = 0.2 *f* (Fig. 5).

**Figure 6:**
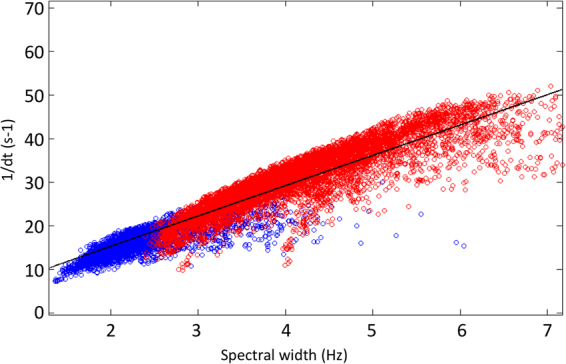
Representation of the covariance of the spectral width with the coherence length. The correlation is 0.86. The product df.dt is equal to 0.3. The black line represents the correlation.

The temporal width is linked to the spectral width and therefore evolves in the opposite direction. This result was confirmed by the time analysis (Fig. 6). All individual patients results can be found in supplementary file ESM1 (frequency domain) and ESM2 (time domain).

### Time compression

We considered the stable unconscious state (30-s) for the six patients and used 1024 Hz datasets for all of them. We computed the ratio of the frequency change for each patient, *r* = *f*_U_/*f*_C_. We then resampled the EEG time series: If we consider the EEG channel time sequence, we resampled the sequence *x* at a rate of *r* times the original sampling rate. The length of the result *y* is *r* times the length of *x*. The result is a compressed ECoG time series during the period of 30.*r s* that is associated with the virtual brain state U_fast_. In other words, 1024 points represented 1 s before rescaling, and after rescaling; the 1024 points represent 1.6 s for example (r = 1.6). We then applied all the analysis that was performed on the real ECoG data to the new time series.

However, this transformation is mathematical and does not conserve energy. To keep the energy constant, one should divide the amplitude by a factor *r*^1/2^ after time compression. The justification of this corrective factor may be found in material and method section.

This correction allows a similarity in the amplitude of the U_Fast_ and C states, but does not change the frequency characteristics. However, we did not compare amplitude because it was subject to frequent electrical impedance changes during the recording: the surgeon pressed the grid or added saline solution to improve conduction and therefore the interpretation for one patient was impossible. After the compression analysis, we obtained the following set of physical quantities: Amplitude, *f*_UFast_, *δf*_UFast_ and *Q*_UFast_. These values were compared with the real conscious state *f*_C_, *δf*_C_ and *Q*_C_.

Our results strongly suggest that there is a scaling factor between the two states (U and C). To demonstrate this hypothesis we considered the EEG trace for the unconscious state (U), and we rescaled time with respect to the frequency ratio *r* that we observed around the transition from GA (*f*_U_) to ROC (*f*_C_) with *r* = *f*_U_/*f*_C_.

We showed that the frequency-shifted spectrum matches the spectrum during the conscious C state. We applied time compression to the six patients in the unconscious state, and we showed that we did find the same spectrum characteristics (Fig. 5-B) for the coherent spectrum. Indeed, the true C state was the conscious state C: (*f*_0_ = 18.8 ± 0.4 Hz, *δf* = 4.4 ± 0.2 Hz, Q = 4.2 ± 0.1) while the U_fast_ state that corresponds to the C state compressed in time was (*f*_0_ = 18.6 ± 0.6 Hz, *δf* = 4.4 ± 0.1 Hz, Q = 4.2 ± 0.1). There was no statistically significant difference between C and U_Fast_ (P = 0.4 for f comparison, P = 1.0 for *δf* comparison, P = 0.8 for Q comparison).

To summarize, we showed that the carrier frequency, FWHM and coherence time change during the transition from unconsciousness to consciousness within the same scaling. After time compression, the U state shares the same characteristics as the C state and, therefore, the two signals can be deduced from each other through a time scaling factor for the coherent part of the EEG signal.

### Mathematical modelling

We hypothesized that the coherent part of the spectrum is due to pulse trains. An envelope and an oscillatory part represent each elementary pulse. An electrical pulse can be modelled with a carrier frequency *f*_0_ and a temporal width τ. We choose a hyperbolic secant (sech) pulse modelisation even if the exact pulse shape is not important for the demonstration. We construct a theoretical ECoG trace formed by the train of sech pulses distributed randomly over time. We then computed the FRIE trace of the conscious state that corresponds to a central frequency of 11 Hz and then compressed the time by introducing mathematically the time scaling factor r and then computed the FRIE for the novel trace.

**Figure 7:**
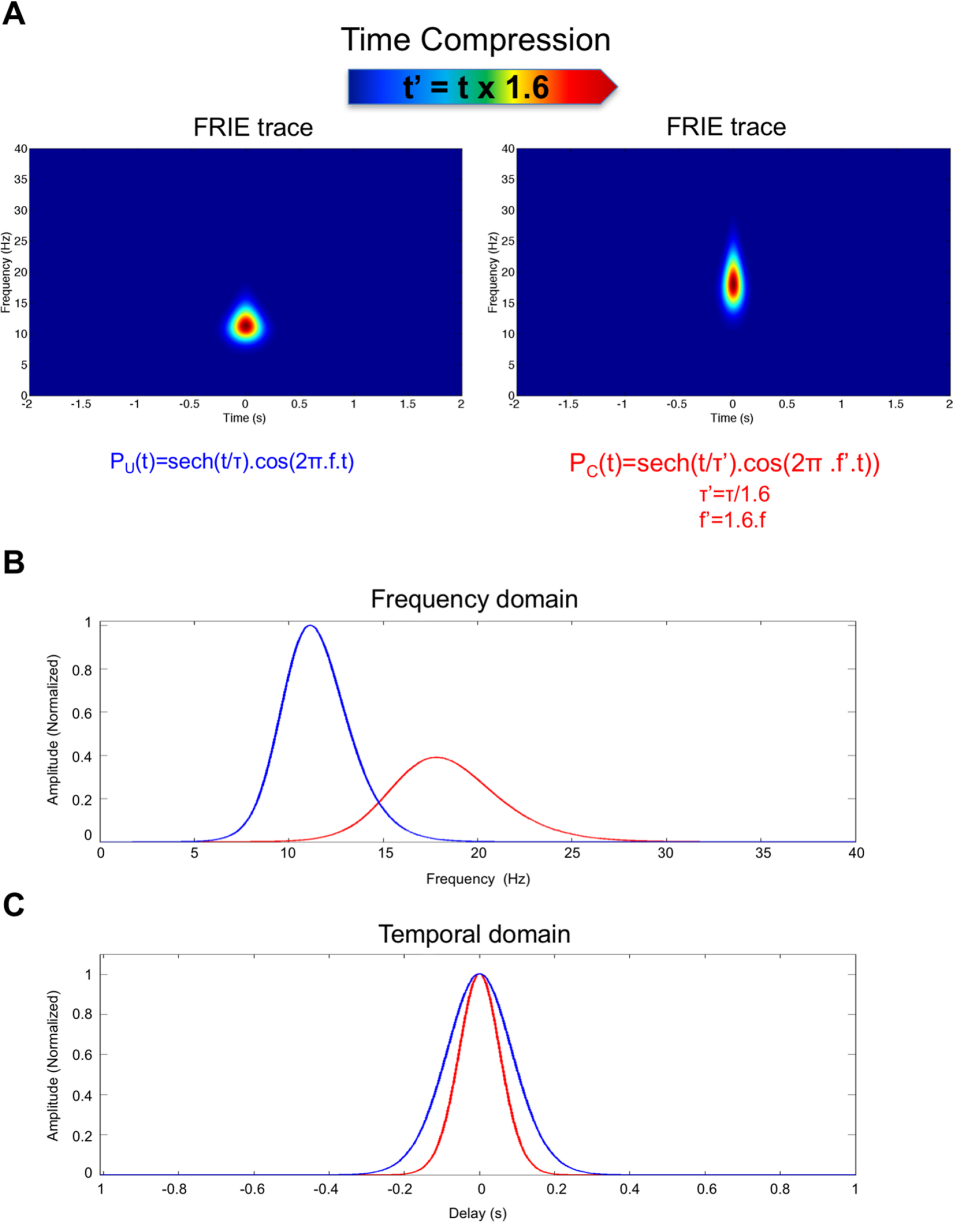
Result of numerical computation of time rescaling of a sech pulse: (**A**) the time frequency analysis shows the same features than Fig. 3: a shift toward high frequency and spectral widening (**B**) associated to a shortening of pulse duration (blue is before time compression and red is after time compression).

The result of the time scaling for the train pulse described by a hyperbolic secant is a shift in frequency and a compression of the pulse duration thus a spectral widening that is exactly what was observed in the ECoG changes (Fig. 3 and Fig. 7).

#### ROC modelisation using Jansen and Rit Model

To understand our observations, we developed a biophysical model of the cortex. The cortex is considered as a medium that can oscillate. Indeed, excitatory neurons are connected to inhibitory neurons that control their activity via feedback and feed forward inhibition^20^. That interaction has been extensively studied and gives rise to oscillations^21–23^.

We hypothesised that an increase in inhibition slows the integration time and lengthens the refractory time, which leads to decreased neural transmission, and therefore a lower frequency oscillation.

**Figure 8:**
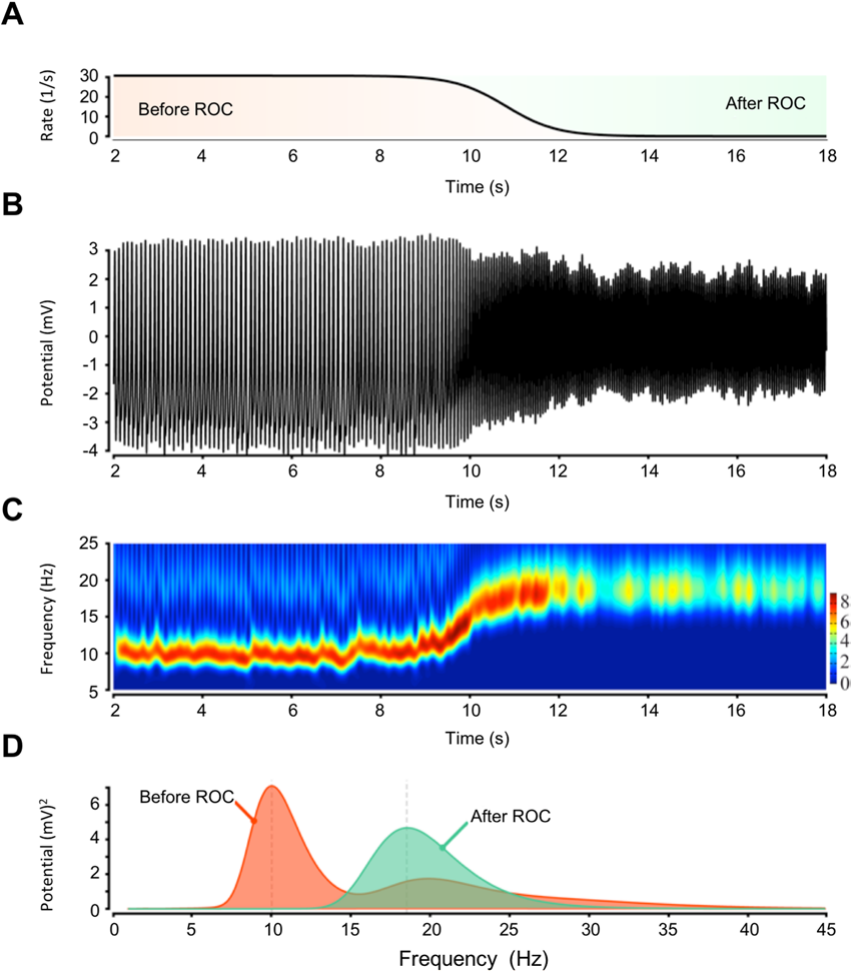
Result of Jansen et Rit Model of cortex under inhibition: Frequency increases with decreasing inhibition (in the model) during the recovery of consciousness (ROC). (**A**) Decrease in the inhibition tone. (**B**–**D**) The dominant rhythmic activity from the alpha into the beta band. (**A**) The inhibitory interneurons are excited before ROC and are recovering back to normal after the ROC. The mean postsynaptic potential of the pyramidal cells is shown in **B** and is transformed in the time-frequency domain in **C**. The frequency spectrum before and after ROC is shown in **D**.

To test this hypothesis, we used a Jansen and Rit neural mass (NM) model of the local network (see Materials and Methods section). We have considered a model of a cerebral area by affecting the inhibition in the considered local simulated circuitry. We assumed over-excited inhibitory interneurons during GA, which recover to a normal baseline after ROC. To describe the drive of the excitation of the inhibitory interneurons in the model (i.e. the extrinsic input), we used the sigmoid function together with its temporal features, which we obtained from the analysis of the ECoG data (see Fig. 4B). In this way, we could reproduce the measured effects on the brain oscillations during ROC (see e.g. Fig. 8). The model predicts that the pyramidal cells in the cerebral cortex are over-inhibited before ROC due to increased activity of the inhibitory interneurons (extrinsic input > 0), which could be due to an external intervention such as intravenous propofol (anesthesia) or internal extracortical intervention (thalamus). ROC was modelled by a return of the inhibitory interneurons’ activity (before ROC) to the baseline (after ROC), which results in accelerated rhythmic activity after ROC.

The experimental results show that there are two characteristic frequencies, a lower frequency around 10 Hz (*f*_U_) and the higher frequency around 18.6 Hz (*f*_C_). (f_c_/f_u_ = 1.86). The latter corresponds to a situation without continuous inhibition and the former corresponds to saturated inhibition. In this computation, spectral FWHM where δ*f*_U_ = 3.42 Hz and δ*f*_c_ = 6.40 Hz in exactly the same ratio f_c_/f_u_ = δf_c_/δf_u_ = 1.86). Therefore, the Jansen and Rit simulation not only reproduces the frequency shift but also the spectral widening.

## Discussion

ECoG data analysis using interferometry clearly shows that the oscillatory behavior that is centered on 10 to 20 Hz depending on the consciousness state is different from the background activity that behaves as a scale-free phenomenon (1/f^m^). Indeed, the oscillatory phenomenon shows a highly coherent structure. It is formally similar to train of electrical pulses. The rescaling process shows that the oscillatory behavior follows a scaling law between the unconscious and the conscious state. This means that the two states may be deduced one from the other by a time rescaling.

The significance of this time rescaling may have a simple physiological interpretation. The oscillatory phenomenon appears as pulse of activity that has a beginning and an end: the oscillation built up and then vanish. The characteristic time of envelope evolution τ_e_ translates the gain and damping of the oscillation^24^.

The oscillation frequency is a function of the speed c of the process that creates it. Indeed, a fast process creates a high frequency. In the case of EEG, the speed may represent the conduction speed of the neural transmission.

The building-damping of the oscillation may be described by the Quality factor of the oscillator Q that represents the number of oscillation contained in a single activity. If one assumes that the oscillator is the same then Q remains constant and therefore τ_e_ should evolves in the same ratio than the central frequency *i*.*e*, if the oscillator is faster then the oscillation damps faster in the same ratio. In the frequency domains this time shortening is translated into spectral widening as it is shown higher.

Therefore, we hypothesize that EEG changes with propofol anesthesia are related to a slowing down of speed processing in cortical networks due to an increase of inhibition by inhibitory interneurons. We simulated the increase of inhibitory tone over a population of neuron using Jansen and Rit model and reproduced the same spectral behavior.

Thus, this work suggests that EEG changes may be understood by the intervention of a high inhibition tone on cortical networks. These networks act as oscillators whose operating central frequency may be tuned by the intervention of an inhibitory tone. During unconsciousness, networks are slowed down and therefore oscillate at a lower frequency (11 Hz) with a longer coherence time. After ROC, the processing is faster (18 Hz and shorter time of coherence).

### Contribution to understanding of anesthesia mechanism

We considered that a neural network might be a system that processes incoming pulses. The system will process inputs according to its inhibition set point.

This description changes the way we analyse the EEG signal. Our approach does not need to separate the EEG signal into frequency bands. The electrical impulses received by cortical neural networks from other networks are very similar in nature to other electrical or light impulses that have been extensively studied in the theory of light pulses, radar or electricity. In the EEG signal, the impulse has a frequency content that straddles between alpha and beta bands: Using this approach, the EEG changes observed during ROC were the result of a change in the processing speed. Therefore, when the signal is faster, the frequency content is switched to higher frequency and the spectrum is wider because the impulse is shorter (uncertainty principle).

A recent model of EEG changes to explain the transition between consciousness and unconsciousness was proposed by Koppel *et al*., who introduced a thalamo-cortical loop to explain strong alpha rhythms during anesthesia^25^. Our model does not require a thalamocortical oscillation to explain EEG changes. Cortico-cortical interactions and increased inhibition in the network are the only hypothesis needed in our model. The thalamus very probably plays a role in controlling or modulating the inhibition, but in our model oscillation is created by the cortex itself in response to an incoming pulse. However, condition of oscillation may be under the control of another anatomic region.

The value of our model is to explain most EEG high frequency changes observed during anesthesia and after ROC:

- Switch from main α band to β band due to decreased inhibition.
- Decreased amplitude of the entire EEG because the spectrum is wider.
- The so-called paradoxical excitation (an increase of amplitude with light anesthesia and stage I of sleep) is linked to the slowing of the process leading to concentrating the energy in a narrower band of frequency.
- Broader spectrum leading to higher spectral entropy^26^. Indeed, the correlate of the shortening of the pulse in time is the widening of the spectrum. The number of states in the frequency domain is, therefore, higher leading to higher entropy. However, the fact that the two states are linked by a simple time dilatation/compression factor indicates that the complexity of the signal is rigorously the same and is not more complex in nature. In our opinion, the higher spectral entropy is an artefact due to the partition of the EEG in frequency bands.

### Mechanisms of unconsciousness

In this work, we studied the changes of ECoG during recovery from anesthesia. We suggest that EEG changes observed during unconsciousness are linked to a higher inhibition tone that slowed down signal processing.

One question is how is the inhibition obtained. In our situation, the high inhibitory tone may be due to a direct pharmacological effect of propofol or an indirect effect by promoting higher inhibition from subcortical structures (thalamus?). The ECoG is two-dimensional and shows only cortical phenomena. However, some of the results fovours the intervention of subcortical structures. Indeed, the unconscious state was remarkably stable in frequency and did not differ between patients. It is unlikely that pharmacological effects are stable without differences across patients. Moreover, a relationship between alpha rhythm and inhibitory processes have been largely demonstrated^27^. The same strong alpha oscillations are obtained naturally during sleep (complete deactivation)^28^ or during selective deactivation of a cerebral area: Occipital areas when eyes are closed (Berger effect), somatosensory cortex^29,30^. For example, when attention is allocated to one visual hemifield, increased alpha power is observed in the ipsilateral visual cortex^31^. Processing time slowing seems to be a way to deactivate a cerebral area. The ubiquity of this EEG pattern highly suggests a role for subcortical structures rather than a local cortical effect.

During anesthesia unconsciousness, the frontal areas show a shift in frequency towards 11 Hz and this is probably the result of subcortical structure inhibitory action. It may suggest that at an early stage, anesthesia provoked the shutting down of the cortex^32^, which is amplified through the cortico-thalamic loop to induce complete loss of consciousness. In other words, the role of propofol is, therefore, to precipitate the brain into a state of high inhibition and prevent it from exiting from that state.

We cannot explain the link between the slowing down of processing and the impairment of cognition and consciousness. Our model does not explain what consciousness is or is not but only the transition from one state to the other regarding electrical changes in the cortex.

### Limitations

#### Unconsciousness and unresponsiveness

A limitation of our work is linked to the above considerations: We still need a more robust definition and theory of consciousness. Indeed, the patients were classified as unconscious when they were unresponsive. It is a pragmatic but certainly too simplified approach. There is a debate about whether unresponsive patients may be conscious^33^. Here, we showed that when patients became responsive there was a modification of the network processing. It is unclear whether the patients were unresponsive because their frontal lobe was inhibited (‘pharmacological adynamical frontal syndrome’) or because they were truly unconscious. Indeed, for some authors, frontal networks are involved in task monitoring and reporting might not be a real anatomic neural correlate of consciousness^34^. However, MRI studies of functional integration during propofol anesthesia showed a breakdown of brain integration (a correlate of consciousness) and also stressed the important role played by parietal and frontal areas in consciousness^14,35^.

However, it is unclear why slowing down signal processing would impair integration. A hypothesis would be impaired communication between distant areas of the brain. Inhibition of frontal to parietal feedback connectivity has been shown to be a neurophysiologic correlate of general anesthesia^36^ and disruption of frontal-parietal communication is a common feature of general anaesthetics related to unconsciousness^37^. A slowing of brain transmission could disrupt cortico-cortical synchronization, an important feature of conscious processing^38^.

#### Other anaesthetics

The results of our study were obtained with the most used intravenous anesthetic, propofol. Other GABA agents share the same effects on EEG pattern (inhaled anaesthetics, benzodiazepine, barbiturates). However, other classes of anesthetics have other receptor targets and therefore different EEG patterns. NMDA agents like xenon, nitrous oxide and ketamine produce very different modifications of the EEG^39,40^. Indeed, these agents fool the usual anesthesia monitors by increasing beta range oscillations and decreasing alpha oscillations but also may increase delta frequencies at high concentration. Our model describes the effect of higher inhibition and may not be extrapolated to NMDA agents.

The difference of EEG pattern between classes of anesthetics suggests that loss of consciousness may result from several mechanisms. Consciousness is a complex situation and any impairment of signal processing even very different in nature may lead to the same endpoint, which is unconsciousness. Any change in the local network ability to respond to its input would disrupt signal processing and integration and impair consciousness.

Another limitation is that ECoG does not give any indication of the dynamics of subcortical structures. The thalamus has likely a role in controlling inhibition of cortical areas and may intervene in slowing down the propagation of the signal. The effects in our model of the interaction between the cortex and subcortical structures have to be tested in future clinical studies using deep brain electrodes.

## Conclusion

We show that the recovery of consciousness corresponds to a transition in the frequency (f, δf) space, which is exactly reproduced by a simple time rescaling. We accelerated the time course of unconscious trace by a factor 1.6 and we showed that the resulting EEG trace matches the conscious state. This rescaling may be explained by a slowing down of speed processing in cortical networks. Therefore, EEG changes during the recovery of consciousness, mainly shift of frequency and spectral widening, may be understood simply as an increase of neural conduction in the frontal area. Our model only relies on cortico-cortical interactions. The effect of subcortical structures on the model must be assessed in further studies.

## Material and Methods

### Clinical study

#### Patients and clinical setting

After Ethics committee (CPP1 Sud Méditerrannée) approval, continuous ECoG signals were acquired in patients who underwent intracranial monitoring during awake surgery for the treatment of cerebral tumors in functional areas of the brain^15,16^. All the study was performed in accordance with ethics committee guidelines. Written informed consent was obtained from all patients before the surgery. EEG recordings were performed initially for clinical purposes: Indeed functional intraoperative mapping in the awake condition by direct electrical stimulation currently represents the gold standard in the surgical treatment of lesions located in eloquent areas^15,41^. Therefore, the ECoG grid covered the left central sulcus and the Sylvian fissure, thus measuring parts of the frontal, parietal and temporal lobes of the left (dominant) hemisphere. No tumour was in the frontal area. We also recorded the ECoG throughout the cessation of anesthesia up to the point of the surgical removal of the tumour. Therefore, we recorded the transition from unconsciousness (state U) to consciousness (state C) directly on the brain surface. ROC was defined by the patient’s response to a simple order and was assessed by the anaesthesiologist before removing the laryngeal mask. Patients were selected from a database according to the following criteria: (1) more than 20 non-noisy frontal electrodes; (2) no tumoural tissues in the vicinity (at least 2 cm) of the frontal area; (3) recording that included the unconscious state; (4) precise timing of ROC.

#### Data acquisition

ECoGs were recorded using a grid of 8 × 8 electrodes (DIXI medical, Besançon, France), where each contact was a 0.5-mm-diameter platinum–iridium cylinder with a 10 mm inter-contact distance. The monopolar recording used a reference that was placed on the forehead of the patient. The EEG acquisition was performed using a Micromed Brain Quick apparatus (Micromed™, Mogliano Veneto, Italy) with a video camera and 64 ECoG channels. The neurosurgeon identified the anatomic location of the tumour, both by direct visual analysis and using a Neuronavigation system (Brainlab AG™, Feldkirchen, Germany) that was set up before the surgery. The original sampling rate was 1024 Hz.

#### Signal processing

Overview: A stated in the main text, ECoG signal contains two different physics: a power law behavior that describes the low (<4 Hz) and the high frequency (>40 Hz) and an oscillating signal that lies in the classical beta and alpha EEG bands.

The processing focuses on the intermediate regime of frequency because it is the most important change that is observed in the signal during the process of ROC. One of the difficulties is to correctly isolate that part of the signal from the power law behavior. We used interferometry to study only the oscillating part of the spectrum that translates into a bell shaped spectrum.

**Figure 9:**
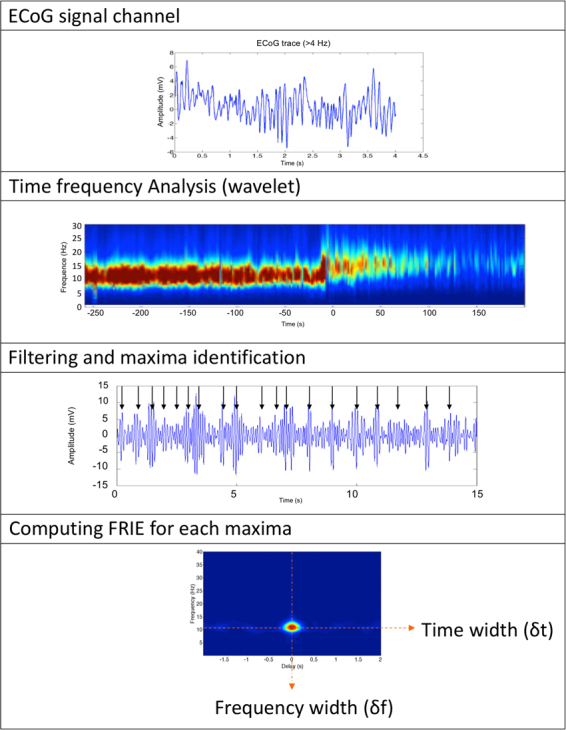
Schematic of the processing of EcoG signals. Step 1 is pre treatment of ECoG traces (removing artefact). Then we computed the time frequency analysis in order to identify maxima of oscillations. For each maximum we computed the interferogram with a sliding window of 1 s.

The schematic of the signal processing is illustrated in Fig. 9. The processing comprises the following steps:

- Computation of a time-frequency analysis for each ECoG channel using wavelet
- Isolation of amplitude maxima of the filtered signal
- Computation of the Frequency resolved interferometry of the ECoG centered on each maximum
- Computation of the central frequency, the spectrum width and the temporal width of the FRIE trace.

Time–frequency study: We performed a time–frequency analysis using wavelets. We selected, by visual inspection, frontal electrodes that were not contaminated with noise and artefacts. The data were down-sampled to 256 Hz to reduce the computation time. We did not down-sample when studying high-frequency (>40 Hz) behavior. Wavelets are appropriate when the signal is non-stationary. Therefore, we computed the wavelet transform of each channel. The wavelet power spectrum is the square of the modulus of the wavelet transform. This method involves convoluting the ECoG signal with a series of ‘daughter’ wavelets, which are time-scaled variants of a ‘mother’ wavelet. We used the Gabor mother wavelet, which is Gaussian, modulated by a complex exponential:

1 $$\Psi (\eta )={e}^{i{\omega }_{o}\eta }{e}^{-\frac{{\eta }^{2}}{{\sigma }^{2}}},$$

where *η* is a dimensionless time parameter, *ω*_0_ is a dimensionless wavelet central frequency that was set to 6 (to satisfy the admissibility criteria), and *σ* is a parameter that controls the exponential rate drop-off. Each channel was decomposed using Gabor wavelets in the time–frequency domain. The frequency resolution was 0.1 Hz.

Time frequency maxima identification: We first made a time–frequency decomposition of all channels. We then computed the envelope of the filtered signal (>4 Hz). As stated in the first section, the ECoG trace appears as a succession of oscillating pulses. We spotted and recorded the maxima of ECoG time-frequency decomposition. To do so we computed the envelope of the ECoG time frequency analysis and then identified the maxima. For each maximum, we computed the FRIE trace 1 s before and after the peak.

FRIE (Frequency resolved Interferometry for ECoG): We used a technique derived from laser optics^42^. This technique was originally designed for ultra fast optical pulses. In the case of ECoG the pulse length is not an issue but the technic is useful to separate the oscillatory signal from the non-oscillating background.

Physics behind a 1/*f*^*m*^ behavior and a bell shaped spectrum are totally different. Indeed a coherent oscillating signal translates in a bell shaped spectrum but never in a 1/ f^m^ spectrum.

A way to distinguish between the two phenomenons is to perform an interferogram. Indeed, arrhythmic sources do not interfere while oscillating sources do.

An oscillating coherent signal has a self-coherence that can be highlighted by computing an interferogram of the signal with itself. Only rhythmic coherent signals interfere with themselves. The computation of an interferogram is based on the same principle of wave physics (Fig. 2): the signal is delayed in time and added to itself for various time delays. The interferogram is the resulting average amplitude of the signal. If the signal is self-coherent, the interferogram displays interference fringes with a visibility that depends on the time of coherence. This coherence time is necessarily greater than the signal period. A perfect sinusoid has an infinite time of coherence, whereas a more realistic oscillatory signal has a vanishing visibility due to a finite coherence time.

This interferogram is a powerful tool for separating oscillating spectral contents from others. We have identified the activity pulses by calculating the ECoG signal envelope filtered between 4 and 40 Hz. For each pulse we have calculated an interferogram. We can therefore obtain for any time step of 2 s an average interferogram.

This interferogram contains all the information necessary to describe the state of the ECoG signal. In order to characterize both frequency content and coherence time duration, we computed a Frequency-Resolved Interferogram of the ECoG Trace (FRIE). It is the time-frequency transform of the interferogram of the ECoG trace. The coherent spectrogram FRIE(*t*, *f*) was described by its amplitude, central carrier frequency *f*, spectral width *δf* and temporal length τ; this characterization is defined as the Full Width at Half Maximum (FWHM) for both frequency and temporal domains. Real spectral and temporal width are closely linked to the FRIE widths by a simple proportionality rule^43^.

Technically, we computed the interference between the interference of the signal *X* with delayed and then computing the mean value:

2 $$C(\tau )=\frac{\langle X,X(\tau )\rangle }{\langle X(t)\rangle \langle X(t)\rangle }=\frac{{\langle (X(t)+X(t+\tau ))\rangle }^{2}}{\langle X(t)\rangle \langle X(t)\rangle }$$

We then computed the time–frequency decomposition of the interference *C*(*τ*) using the same analysis as in the previous paragraph. We obtained a trace FRIE(*τ*, *f*), which represents the decomposition of the interference pattern in the delay–frequency domain. It appears as a two-dimensional bell-shaped curve with a spectral and temporal width.

The spectral and time widths are not exactly the real spectral and time with but they are linked to the real width by a proportionality rule. The link between real spectrum and coherent spectrum is well studied for light pulses and depends on the chosen description for the pulse. There is a correspondence between true spectral (resp. time) FWHM δ_s_ and measured δf using interferogram according to:

3 $${\rm{\delta }}{{\rm{f}}}_{{\rm{s}}}={\rm{k}}.{\rm{\delta }}{\rm{f}}$$

where k is a constant that depends on the assumed pulse shape (for example k = 0.56 for sech pulse and for 0.84 Gaussian shaped pulse)^43^.

We choose to fit the bell shaped spectral and time pulse by a sech pulse because it produced the best fit. However, the pulse choice is not critical.

We computed the central frequency of the pulse *f*_0_, the spectral width *δf* and the temporal width *δt*, which is the coherence length of the signal. We computed for each patient and each electrode the spectral state defined by its central frequency *f*_0_ and FWHM *δf*. We averaged these physical quantities for each electrode and for 20 s of stable unconscious or conscious state.

For each electrode, we also computed the ratio *Q* defined by:

4 $$Q=\frac{f}{\delta f}.$$

Frequency change kinetic study: We computed a mean interferogramm for every patient and each channel. For each patient, we averaged this interferogram every 2 s time step and over all electrodes. We obtained an averaged interferogram evolution with time. We isolated the central frequency and mean width. Therefore, we obtained the evolution of the mean central frequency and FWHM over time. This evolution is a sigmoid that has two states. We fitted the time evolution of the central frequency by a sigmoidal function:

5 $$f(t)={f}_{1}+\frac{{f}_{2}-{f}_{1}}{1+{10}^{r({t}_{0}-t)}}.$$

This equation reflects the evolution of the frequency *f* with time (*t*). The parameters are the frequency before and after the ROC, *f*_1_ and *f*_2_, the centered time of the ROC, *t*_0_, and the slope parameter, *r*, in determining the transition. The slope of the sigmoid, namely, *r*, was expressed in s^−1^. We obtained the two states’ amplitudes *f*_1_ and *f*_2_ and the slope of the change. *f*_1_ and *f*_2_ are slightly different from the frequency obtained above because of spectral jitter, which causes imprecision and spectral widening. However, this method allows time evolution monitoring and kinetic characterisation.

The characteristic transition time Δ*T* was defined as the time needed for the transition from (1 + *x*).*f*_1_ to (1 – *x*).*f*_2_. *x* was set to 5%.

6 $$\Delta T=\frac{1}{r}\,\mathrm{log}(\frac{({f}_{2}-(1+x){f}_{1})((1-x){f}_{2}-{f}_{1})}{{x}^{2}{f}_{1}{f}_{2}})$$

Time compression: We considered the stable unconscious state (200 s) for the six patients and used 1024 Hz datasets for all of them. We computed the ratio of the frequency change for each patient, *r* = *f*_U_/*f*_C_. We then resampled the EEG time series: If we consider the EEG channel time sequence, we resampled the sequence *x* at a rate of *r* times the original sampling rate. The length of the result *y* is *r* times the length of *x*. In other words, 1024 points represented 1 s before rescaling, and after rescaling, the 1024 points represent 1.8 s. We then applied all of the analysis that was performed on the real ECoG data to the new time series.

However, this transformation is mathematical and does not conserve energy. We applied a corrective factor to the wavelet transform amplitude, namely, *r*^1/2^.

Indeed, if we consider *S*(*t*), an ECoG channel signal, its energy may be written as:

7 $$E={\int }_{0}^{\infty }{|S(t)|}^{2}dt.$$

In the same manner, the energy after time compression keeps the modulus constant but changes the time to *rt*:

8 $$E^{\prime} ={\int }_{0}^{\infty }{|S(rt)|}^{2}d(rt).$$

After the variable change *u* = *rt*, we obtain:

9 $$E\text{'}=r{\int }_{0}^{\infty }{|S(u)|}^{2}d(u)=r\,.\,E.$$

To keep the energy constant, one should divide the amplitude by a factor *r*^1/2^ after time compression.

This correction allows a similarity in the amplitude of the U_Fast_ and C states, but does not change the frequency characteristics. However, we did not compare amplitude because it was subject to frequent electrical impedance changes during the recording: the surgeon pressed the grid or added saline solution to improve conduction and therefore the interpretation for one patient was impossible. Nevertheless, correspondence between the two states C and U_Fast_ was good for five patients (ESM 1, supplementary files).

We obtained the following set of physical quantities: Amplitude, *f*_UFast_, *δf*_UFast_ and *Q*_UFast_. These values were compared with the real conscious state *f*_C_, *δf*_C_ and *Q*_C_.

Statistics: All parameters are expressed as mean ± standard deviation. Statistical significance was set at *p* < 0.05 for all statistical tests. Tests were *t*-tests or paired *t*-tests depending on measurements (see each paragraph). Statistical analysis was performed using MATLAB software (MathWorks, Natick, MA, USA).

## Theoretical Study

### Mathematical Model

We hypothesized that the coherent part of the spectrum is due to pulse trains. Each elementary pulse may be represented by an envelope and an oscillatory part.

Indeed, an electrical pulse can be modelled with a carrier frequency *f*_0_ and a temporal width. A simple description is a hyperbolic secant pulse in the time domain, which is described as follows:

10 $$E(t)=\,\mathrm{Sech}(\frac{t}{\tau })\cos (2{\rm{\pi }}{f}_{0}t)$$

where *t* is the time, *τ* expresses the duration of the pulse, and *f*_0_ is the carrier frequency. Sech is the hyperbolic secant. It is a bell-shaped curve widely used in telecommunication^44^.

The pulse defined by (3) has a spectral content due to its finite duration that can be expressed by its Fourier transform:

11 $$\tilde{E}(\nu )=\,\mathrm{Sech}(\frac{f-{f}_{0}}{\delta f}),$$

where *f* is the frequency, *δf* is the pulse spectral width and *f*_0_ is the carrier frequency. Here, *δf* and *τ* are related, and the product *δf*.*τ* is a constant., the coherent unity is a pulse with a finite time length and frequency extension.

There are no a priori reasons why f_0_ and τ (or *δf*.) change in the same proportion.

The factor *Q* = *f*_0_/*δf* represents the number of visible oscillation into the pulse. In oscillators theory, it is called the quality factor of the oscillation. We showed that this factor remains constant during the transition between U and C. This means that there are the same numbers of oscillation during the electrical pulses in the U and C states. Thus the pulse in the C state is a compressed version of the U state pulse. This compression was confirmed by the time scaling experiment.

The result of the time scaling for the pulse described in (10) is a shift in frequency and a compression of the pulse duration thus a spectral widening that is exactly what was observed in the EEG changes (Fig. 3 and Fig. 7).

Indeed, the time scaling (t’ = t.1.6) changes equation 10 to:

12 $$E(t)=\,\mathrm{Sech}(\frac{t}{\tau \text{'}})\cos (2{\rm{\pi }}f{\text{'}}_{0}t)$$

where:

13 $$\tau ^{\prime} =\frac{\tau }{1.6}$$

Therefore that translate into the frequency domain by:

14 $$\delta f^{\prime} =\mathrm{1.6.}\delta f$$

and:

15 $${f}_{0}^{\text{'}}=\mathrm{1.6.}{f}_{0}$$

### Jansen and Rit Model, Generative model

The Jansen and Rit model (i.e. the local circuit of pyramidal cells, excitatory and inhibitory interneurons) was scaled to perform a slow rhythm (approximately 10 Hz in Fig. 4) under an over-excitation of inhibitory interneurons (extrinsic input of ~30 s^−1^ in Fig. 4). In this regime, the model is monostable. The transition from the lower (before ROC) to the higher frequencies (after ROC) was caused in the model by a change in the extrinsic excitation of the inhibitory interneurons. Note that ‘extrinsic’ here means anything that is independent and, thus, not generated by the cortex or the three Neural Masses (NM) in the model (i.e. pyramidal cells, excitatory and inhibitory interneurons). The extrinsic change creates the slow variable in the model (see panel A in Fig. 4). In contrast to this slow change, the interactions between the three NMs are rather fast (e.g. alpha/beta oscillations in panels B to D in Fig. 4). The model was directly informed about the frequency transition over time by the fit of the evolution with a sigmoid function. This function was then used to drive the extrinsic input at the inhibitory interneurons. Before the ROC, this input (in Fig. 4 with an incoming firing rate of approximately 30 s^−1^) caused an additional mean postsynaptic potential at the inhibitory interneurons (of approximately 1 mV in Fig. 4). The extrinsic input is simply zero after the ROC. The over-excited inhibitory interneurons caused a monostable slow rhythm that speeds up if the inhibition in the local circuitry goes back to the baseline.

A generative model for brain measurements such as M/EEG can be specified by two separate systems: the state system *q*, which explains the usually hidden neuronal states **x** (e.g. the mean postsynaptic potentials (PSPs) of the neuronal populations that potentially generate the M/EEG); and the observer system *k*, which relates the neuronal states to the measurements **s**:

16 $$L(\frac{d}{dt}){\boldsymbol{x}}=q({\boldsymbol{x}},{{\boldsymbol{p}}}_{{\boldsymbol{x}}}),$$

and

17 $${\bf{s}}=k({\bf{x}},{{\bf{p}}}_{{\bf{s}}}),$$

where *L*(d/d*t*) is a temporal differentiation operator, and **p**_**x**_ and **p**_**s**_ parameterize the state and the observer systems, respectively. For the state system *q*, we used a local network model of the NMs of a cerebral area. For the observer system *k*, we used a simple linear relationship, where **s** describes the ECoG that is directly recorded on the cortical surface, and we simply considered a single area (i.e. a source).

To describe the dynamics of the cortical area, we used the model by Jansen and Rit^45^. In this model, the local network consists of three interacting NMs: pyramidal cells (PCs: NM 3) with feedback loops that are mediated by excitatory and inhibitory interneurons (EINs and IINs: NMs 1 and 2, respectively). This basic circuit has been described in previous studies. Note that the connections can also be modelled dynamically; for example, by considering the transmission delays^46–48^. Here, we assume that there are connections within a single area that result in transmission times that are shorter than the characteristic (dendritic) time constant *τ*_e_ and are thus negligible. Therefore, it is sufficient to describe the feedback connection by a gain constant.

With this model, the mean neuronal states can be described by a system of four non-linearly coupled second-order ordinary differential equations:

- pyramidal cells (NM 3) to excitatory interneurons (NM 1)
  18 $$\begin{array}{rcl}\frac{d{x}_{13}(t)}{dt} & = & {y}_{13}(t),\\ {\tau }_{e}\frac{d{y}_{13}(t)}{dt} & = & {H}_{e}({m}_{1{\rm{E}}}(t)+{c}_{13}{\rm{S}}({x}_{31}(t)+{x}_{32}(t)))-2{y}_{13}(t)-{x}_{13}(t)/{\tau }_{{\rm{e}}},\end{array}$$
- pyramidal cells (NM 3) to inhibitory interneurons (NM 2)
  19 $$\begin{array}{rcl}\frac{d{x}_{23}(t)}{dt} & = & {y}_{23}(t),\\ {\tau }_{e}\frac{d{y}_{23}(t)}{dt} & = & {H}_{e}\,({m}_{2{\rm{E}}}(t)+{c}_{23}{\rm{S}}({x}_{31}(t)+{x}_{32}(t)))-2\,{y}_{23}(t)-{x}_{23}(t)/{\tau }_{{\rm{e}}},\end{array}$$
- excitatory interneurons (NM 1) to pyramidal cells (NM 3)
  20 $$\begin{array}{rcl}\frac{d{x}_{31}(t)}{dt} & = & {y}_{31}(t),\\ {\tau }_{e}\frac{d{y}_{31}(t)}{dt} & = & {H}_{e}\,({m}_{3{\rm{E}}}(t)+{c}_{31}{\rm{S}}({x}_{13}(t)))-2\,{y}_{31}(t)-{x}_{31}(t)/{\tau }_{{\rm{e}}},\end{array}$$
- inhibitory interneurons (NM 2) to pyramidal cells (NM 3)

21 $$\begin{array}{rcl}\frac{d{x}_{32}(t)}{dt} & = & {y}_{32}(t)\\ {\tau }_{i}\frac{d{y}_{32}(t)}{dt} & = & {H}_{i}\,({m}_{3{\rm{E}}}(t)+{c}_{32}{\rm{S}}({x}_{23}(t)))-2\,{y}_{32}(t)-{x}_{32}(t)/{\tau }_{{\rm{i}}},\end{array}$$

where the state vector **x** = (*x*_13_, *x*_23_, *x*_31_, *x*_32_, *y*_13_, *y*_23_, *y*_31_, *y*_32_)^T^ contains the mean PSPs *x*_*ba*_ and the currents *y*_*ba*_ at NM *b* caused by NM *a*. The extrinsic afferents *E* that are projected to NM *b* are denoted by *m*_*b*E_(*t*). The average synaptic gains or the average numbers of synaptic contacts established between NMs *a* and *b* are represented by the constants *c*_*ba*_. Furthermore, *τ*_e_ and *τ*_i_ are the excitatory and inhibitory dendritic time constants, and *H*_e_ and *H*_i_ are the excitatory and inhibitory synaptic gains. The transfer function *S*(*x*_*b*_) that converts the mean PSP *x*_*b*_(*t*) = Σ_*a*_ *x*_*ba*_(*t*) (i.e. the potential at the axonal hillock) to the mean firing rate, in other words, *m*_*b*_(*t*), is taken to have a sigmoidal shape:

22 $$S({x}_{b})=\frac{2{e}_{0}}{1+{10}^{rs({x}_{0}-{x}_{b})}},$$

where 2*e*_0_ is the maximum firing rate, *x*_0_ represents the mean, and *r*_*S*_ represents the variance of the firing threshold within an NM.

Jansen and Rit proposed a specific parameter configuration for generating alpha oscillations based on a thorough analysis of the literature^45^. However, the normalization of time *t* and the potentials *x*_*ba*_(*t*) in the system (Eqs 18 to 21) with respect to the excitatory dendritic time constant *τ*_e_ and the sigmoid slope *r*_*S*_ allow for the scaling of the dynamics to other frequencies or potential ranges (i.e. conserving the bifurcations). The conditions for this circumstance are the constant products of H^*^_e_ τ^*^_e_ = 32.50 μV s and H^*^_i_ τ^*^_i_ = −440 μV s^49^, where H^*^_e,i_ and τ^*^_e,i_ are the values given by Jansen and Rit^39^. To generate a specific rhythm, we provided the dendritic time constants *τ*_e_ and *τ*_i_, which follow Spiegler *et al*.^21^, and we determined the synaptic gains *H*_e_ and *H*_i_ by *H*_e_ = (H^*^_e_ τ^*^_e_)/*τ*_e_ and *H*_i_ = (H^*^_i_ τ^*^_i_). The regime with two co-existing rhythms (i.e. two stable limit cycles), regime V in Spiegler *et al*.^49^; see Figs 2 and 3), was chosen to describe the activity during GA and, thus, before ROC (*t* < *t*_0_). For this purpose, the firing rate via the extrinsic afferents to the NM of the inhibitory interneurons was set to *m*_2T_(*t* < *t*_0_) = H^*^_e_ τ^*^_e_ 30.67 s^−1^/(*H*_e_ *τ*_e_), and the extrinsic input at the NM of the pyramidal cells was set to <*m*_3T_(*t*) > _*t*_ = H^*^_e_ τ^*^_e_ 245 s^−1^/(*H*_e_ *τ*_e_) with a standard deviation of H^*^_e_ τ^*^_e_ 15 s^−1^/(*H*_e_ *τ*_e_) (Gaussian noise). The extrinsic input at the NM of excitatory interneurons is constantly zero, *m*_1T_ = 0. The state was initialized in the unexcited state (e.g. the lower state of the equilibrium manifold, see Fig. 2 in Spiegler *et al*., 2010), in such a way that the system performs slow oscillations (i.e. slow with respect to the other rhythm). To obtain a frequency of approximately 10 Hz for this rhythm, such as in the example shown in Fig. 4, we chose the dendritic time constants of *τ*_e_ = 6.8 ms and *τ*_i_ = 26 *τ*_e_/17. Finally, to describe the acceleration of the rhythmic activity during the ROC, the extrinsic input to the NM of the inhibitory interneurons was decreased monotonically to zero over time, following the form of a sigmoid:

23 $${m}_{2T}(t)=\frac{{m}_{0}}{1+{10}^{r(t-{t}_{0})}},$$

with the maximum input *m*_0_ = *m*_2T_ (*t* < *t*_0_) before ROC at approximately *t*_0_ and with a transition described by the slope parameter *r*. For the example in Fig. 4, the slope parameter was *r* = 7/4 s^−1^. With weaker extrinsic excitation of the inhibitory interneurons, the two co-existing limit cycles in the circuit merge, and the frequency increases. After ROC, the model performs a faster rhythm, in other words, the limit cycle is generated by two supercritical Andronov–Hopf bifurcations in the upper branch of the equilibrium manifold (see regime III in Fig. 2 in Spiegler *et al*.).

## Electronic supplementary material

ESM1

ESM2

ESM3
