# Supplementary material for: Time rescaling reproduces EEG behavior during transition from propofol anesthesia-induced unconsciousness to consciousness
Source: Sci Rep. 2018 Apr 16;8:6015. doi: 10.1038/s41598-018-24405-z (PMC5902625; doi:10.1038/s41598-018-24405-z)
Supplement: Supplementary file 1 — ESM1 [file 41598_2018_24405_MOESM1_ESM.pdf]

Time rescaling reproduces EEG behavior during transition from propofol anesthesia-induced unconsciousness to consciousness

S. Boussen, A. Spiegler, C. Benar, M. Carrère, F. Bartolomei, P. Metellus, R. Voituriez, L. Velly, N. Bruder, A. Trébuchon

ESM 1: Patients' individual results

State /	Patient n°	1	2	3	4	5	6
**Number of electrodes**		**40**	**35**	**34**	**40**	**21**	**26**
**U**	f_U (Hz)	**11.3 ± 0.2**	**12.0 ± 0.2**	**11.3 ± 0.4**	**12.7 ± 0.6**	**11.0 ± 0.3**	**11.3 ± 0.1**
**U**	δfU (Hz)	**2.8 ± 0.1**	**2.8 ± 0.2**	**2.6 ± 0.2**	**2.9 ± 0.3**	**2.6 ± 0.1**	**2.6 ± 0.1**
**U**	Q	**4.1 ± 0.2**	**4.3 ± 0.2**	**4.3 ± 0.2**	**4.3 ± 0.3**	**4.1 ± 0.2**	**4.3 ±0.1**
**U**	Amplitude (A.U)	**0.9 ± 0.4**	**1.4 ± 0.6**	**1.1 ± 0.4**	**0.6 ± 0.3**	**1.2 ± 0.5**	**0.5 ± 0.1**
**C**	f_U (Hz)	**18.2 ± 1.1**	**18.5 ± 1.5**	**18.9 ± 1.0**	**19.1 ± 1.9**	**19.1 ± 1.4**	**19.2 ±1.5**
**C**	δfU (Hz)	**4.4 ±0.3**	**4.3 ± 0.3**	**4.1 ± 0.2**	**4.4 ± 0.3**	**4.4 ± 0.2**	**4.6 ±0.3**
**C**	Q	**4.1 ± 0.2**	**4.3 ± 0.2**	**4.6 ± 0.2**	**4.3 ± 0.4**	**4.3 ± 0.3**	**4.2 ± 0.3**
**C**	Amplitude (A.U)	**0.2 ± 0.1**	**0.4 ± 0.2**	**1.0 ± 0.7**	**0.9 ± 1.0**	**0.4 ± 0.2**	**0.2 ± 0.1**
**C/U**	f_C/ f_U	**1.60± 0.1**	**1.55± 0.1**	**1.68± 0.1**	**1.52± 0.2**	**1.72± 0.1**	**1.70 ± 0.1**
**C/U**	$\delta f_C / \delta f_U$	**1.61± 0.1**	**1.56± 0.1**	**1.56± 0.1**	**1.53± 0.2**	**1.66± 0.1**	**1.72 ± 0.1**
**U_{Fast}**	f_U (Hz)	**17.9 ± 0.7**	**18.1 ± 1.0**	**18.3 ± 0.7**	**19.0 ± 1.0**	**18.9 ± 0.6**	**19.3 ±0.2**
**U_{Fast}**	δfU (Hz)	**4.4 ±0.2**	**4.3 ± 0.3**	**4.4 ± 0.2**	**4.3 ± 0.4**	**4.6 ± 0.2**	**4.2 ±0.1**
**U_{Fast}**	Q	**4.1 ± 0.2**	**4.2 ± 0.2**	**4.2 ± 0.2**	**4.5 ± 0.4**	**4.1 ± 0.2**	**4.2 ± 0.3**
**U_{Fast}**	Amplitude (A.U)	**0.25 ± 0.1**	**0.4 ± 0.2**	**0.4 ± 0.1**	**0.8 ± 0.8**	**0.4 ± 0.2**	**0.15 ± 0.05**
**C/U_{Fast}**	f_C/ f_{UFast}	**1.58± 0.04**	**1.51± 0.05**	**1.62± 0.03**	**1.50± 0.05**	**1.71± 0.04**	**1.71± 0.05**
**C/U_{Fast}**	$\delta f_C / \delta f_{UFast}$	**1.60± 0.07**	**1.54± 0.1**	**1.66± 0.08**	**1.45± 0.1**	**1.72± 0.04**	**1.61± 0.05**
