# Supplementary material for: Time rescaling reproduces EEG behavior during transition from propofol anesthesia-induced unconsciousness to consciousness
Source: Sci Rep. 2018 Apr 16;8:6015. doi: 10.1038/s41598-018-24405-z (PMC5902625; doi:10.1038/s41598-018-24405-z)
Supplement: Supplementary file 2 — ESM2 [file 41598_2018_24405_MOESM2_ESM.pdf]

Time rescaling reproduces EEG behavior during transition from propofol anesthesia-induced unconsciousness to consciousness

S. Boussen, A. Spiegler, C. Benar, M. Carrère, F. Bartolomei, P. Metellus, R. Voituriez, L. Velly, N. Bruder, A. Trébuchon

ESM 2: Transition Time and Coherent Time change during recovery of consciousness

State / Patient n°	1	2	3	4	5	6
**Transition rate (s^{-1})**	0.021 **±0.003**	**0.0056 ± 0.0008**	**0.006 ± 0.001**	**0.0026 ± 0.0004**	**0.004 ± 0.001**	**0.012 ± 0.003**
**Transition Time (s)**	**77**	**295**	**284**	**673**	**453**	**130**
**Time Width (ms)**						
**U**	**154 ± 47**	**151 ± 48**	**145 ± 41**	**136 ± 38**	**150± 41**	**149 ± 42**
**C**	**91 ± 28**	**99 ± 23**	**99 ± 38**	**85 ±20**	**94 ± 31**	**93 ± 27**
**U/C**	**1.69**	**1.53**	**1.46**	**1.60**	**1.60**	**1.60**
